# Can Emotional and Behavioral Dysregulation in Youth Be Decoded from Functional Neuroimaging?

**DOI:** 10.1371/journal.pone.0117603

**Published:** 2016-01-05

**Authors:** Liana C. L. Portugal, Maria João Rosa, Anil Rao, Genna Bebko, Michele A. Bertocci, Amanda K. Hinze, Lisa Bonar, Jorge R. C. Almeida, Susan B. Perlman, Amelia Versace, Claudiu Schirda, Michael Travis, Mary Kay Gill, Christine Demeter, Vaibhav A. Diwadkar, Gary Ciuffetelli, Eric Rodriguez, Erika E. Forbes, Jeffrey L. Sunshine, Scott K. Holland, Robert A. Kowatch, Boris Birmaher, David Axelson, Sarah M. Horwitz, Eugene L. Arnold, Mary A. Fristad, Eric A. Youngstrom, Robert L. Findling, Mirtes Pereira, Leticia Oliveira, Mary L. Phillips, Janaina Mourao-Miranda

**Affiliations:** 1 Department of Computer Science, Centre for Computational Statistics and Machine Learning, University College London, London, United Kingdom; 2 Department of Physiology and Pharmacology, Federal Fluminense University, Niteroi, Brazil; 3 Department of Psychiatry, Western Psychiatric Institute and Clinic, University of Pittsburgh Medical Center, University of Pittsburgh, Pittsburgh, United States of America; 4 University Hospitals Case Medical Center/Case Western Reserve University, Cleveland, United States of America; 5 Department of Psychiatry and Behavioral Neuroscience, Wayne State University, Detroit, United States of America; 6 Cincinnati Children’s Hospital Medical Center, University of Cincinnati, Cincinnati, United States of America; 7 The Research Institute at Nationwide Children’s Hospital, Columbus, United States of America; 8 Department of Child Psychiatry, New York University School of Medicine, New York, United States of America; 9 Department of Psychiatry, Ohio State University, Columbus, United States of America; 10 Department of Psychology, University of North Carolina at Chapel Hill, Chapel Hill, United States of America; 11 Department of Psychiatry, Johns Hopkins University, Baltimore, United States of America; 12 Department of Psychological Medicine, Cardiff University, Cardiff, United Kingdom; University Medical Center Goettingen, GERMANY

## Abstract

**Introduction:**

High comorbidity among pediatric disorders characterized by behavioral and emotional dysregulation poses problems for diagnosis and treatment, and suggests that these disorders may be better conceptualized as dimensions of abnormal behaviors. Furthermore, identifying neuroimaging biomarkers related to dimensional measures of behavior may provide targets to guide individualized treatment. We aimed to use functional neuroimaging and pattern regression techniques to determine whether patterns of brain activity could accurately decode individual-level severity on a dimensional scale measuring behavioural and emotional dysregulation at two different time points.

**Methods:**

A sample of fifty-seven youth (mean age: 14.5 years; 32 males) was selected from a multi-site study of youth with parent-reported behavioral and emotional dysregulation. Participants performed a block-design reward paradigm during functional Magnetic Resonance Imaging (fMRI). Pattern regression analyses consisted of Relevance Vector Regression (RVR) and two cross-validation strategies implemented in the Pattern Recognition for Neuroimaging toolbox (PRoNTo). Medication was treated as a binary confounding variable. Decoded and actual clinical scores were compared using Pearson’s correlation coefficient (r) and mean squared error (MSE) to evaluate the models. Permutation test was applied to estimate significance levels.

**Results:**

Relevance Vector Regression identified patterns of neural activity associated with symptoms of behavioral and emotional dysregulation at the initial study screen and close to the fMRI scanning session. The correlation and the mean squared error between actual and decoded symptoms were significant at the initial study screen and close to the fMRI scanning session. However, after controlling for potential medication effects, results remained significant only for decoding symptoms at the initial study screen. Neural regions with the highest contribution to the pattern regression model included cerebellum, sensory-motor and fronto-limbic areas.

**Conclusions:**

The combination of pattern regression models and neuroimaging can help to determine the severity of behavioral and emotional dysregulation in youth at different time points.

## Introduction

High comorbidity rates among pediatric disorders characterized by behavioral and emotional dysregulation, including bipolar spectrum disorders (BPSD), major depressive disorder, attention deficit/hyperactivity disorder (ADHD), disruptive disorders, and anxiety disorders [1–5], create diagnostic, treatment and research challenges [6]. Adopting the dimensional approach advocated by the National Institute of Mental Health Research Domain Criteria (RDoC) in the study of these pediatric disorders may help identify dimensions of behavioral and emotional dysregulation in youth that cut across categorically-defined diagnoses [7]. In parallel, identifying biomarkers reflecting pathophysiological processes associated with these dimensions of behaviour has the potential to provide biologically-relevant targets that can guide treatment choice and novel treatment development [8,9]. While studies using neuroimaging techniques can identify these biomarkers, the conventional approach in most neuroimaging studies has been to use univariate analyses to compare groups of psychiatrically unwell and healthy individuals. This approach has been used for making regionally specific inferences about abnormalities in brain function and structure that may be associated with a given psychiatric illness [10,11]. Furthermore, this approach has also been used for finding associations between signal of individual regions and proximal clinical measures [12,13]. A major limitation of conventional univariate statistical analysis, however, is that they describe differences at the group level and do not enable decisions *at the individual level*, which is of more limited use in clinical practice, where physicians need to make decisions about individuals.

Pattern recognition is a subfield of machine learning, which uses computer-based techniques to automatically discover regularities in the data, i.e. patterns [14]. Discovery of such regularities in existing datasets can then be used to make predictions for new datasets. For example, pattern recognition has been previously applied to identify patterns in structural or functional neuroimaging datasets associated with a given psychiatric illness, then to classify an independent series of individuals, case by case, into different illness categories, based on their individual-level pattern of structural or functional neuroimaging data [15].

Neuroimaging and pattern recognition techniques can also be used to identify relationships between patterns of brain structure or activity and continuous measures of behaviour or symptoms. Such information can then be used to decode individual-level scores on behavioral measures in independent series of individuals [16,17]. These techniques are promising for identifying neurobiological measures that can predict scores on a given dimension, for example dimensional measures of behavioral or emotional dysregulation, in youth, but their potential here remains unrealized. It should be noted that in the context of pattern regression analyses based on neuroimaging data, the term “predict” means that once the pattern regression model learns a relationship between patterns of brain activation and a clinical score, given a brain scan of a new individual, the model can “predict” or “decode” the clinical score of the new individual.

The Longitudinal Assessment of Manic Symptoms (LAMS) study is a multi-site study of youth with a variety of behavioral and emotional dysregulation diagnoses (please see S1 Text) that aims to assess relationships among the longitudinal course of symptoms, clinical characteristics, and functional outcomes in these youth. LAMS assessed dysregulation with the Parent General Behavior Inventory-10 Item Mania Scale (PGBI-10M), which is a parent self-report dimensional measure of behavioral and emotional dysregulation in youth. This scale includes measurement of manic-like behaviors associated with difficulty regulating positive mood and energy [18,19]. PGBI-10M scores have also been positively and significantly associated with higher scores on the Drive and Fun-seeking subscales of the Behavioral Activation Scale (BAS; r = 0.33 and r = 0.25, respectively, p<0.05) in 816 youth seeking outpatient services, suggesting that PGBI-10M also captures information regarding reward sensitivity in youth (Youngstrom, personal communication). This is an important feature of the PGBI-10M scale, given that sensitivity to rewarding events is considered an important dimension of psychopathology, especially affective psychopathology [20]. Furthermore, those LAMS youth with higher PGBI-10M scores had worse overall functioning and higher rates not only of bipolar spectrum disorders, but also of a variety of other disorders, including ADHD, disruptive disorders, other mood disorders, and anxiety disorders [21]. These findings suggest that the PGBI-10M scale may measure a dimension of behavioral and emotional dysregulation and reward sensitivity that cuts across different diagnostic categories.

The second 5-year phase of LAMS, LAMS2, includes neuroimaging on a subgroup of youth from the original 5-year LAMS study (LAMS1), and provides a unique opportunity to examine whether individual PGBI-10M scores can be decoded from individual-level measures of neural activity in LAMS2 youth.

The aim of the present study was thus to use functional neuroimaging and pattern regression analysis to determine, as a proof of concept, whether PGBI-10M scores at different time points (initial study screen and close to the fMRI scanning session) could be decoded from patterns of brain activity during a reward-processing task in individual LAMS2 youth. This approach has the potential to identify important biomarkers not only to help determine severity of behavioral and emotional dysregulation at the individual level, but also to pave the way forward for future studies using a combination of pattern recognition and neuroimaging to identify individual-level patterns of neural function that predict future clinical outcomes in behaviorally and emotionally dysregulated youth. Given the association between PGBI-10M scores and reward sensitivity, we employed a reward-processing task previously employed in neuroimaging studies in youth [20,22] to focus our study on reward neural circuitry.

## Materials and Methods

### Participants

To investigate whether individual symptom severity could be decoded from whole-brain pattern of activity, we revisited a LAMS2 dataset that was originally analyzed using conventional univariate statistical analysis [12]. A sample of LAMS2 youth from three of the LAMS sites (n = 107; 10–17 years; M = 13.4 and SD = 1.99; chosen to represent both genders, and the range of PGBI-10M scores in each site) participated in this neuroimaging study: Case Western Reserve University (CWRU); Cincinnati Children’s Hospital (CCH); and University of Pittsburgh Medical Center/Western Psychiatric Institute and Clinic (UPMC). Prior to study participation, written informed consent from parents/guardians and written informed assent from children were obtained. The LAMS study received ethical approval from the Institute Review Board (IRB) of each of the participating universities (IRB number REN13060197 / IRB503094). Participants received monetary compensation and a framed picture of the structural neuroimaging scan.

Participants were excluded from the LAMS2 neuroimaging study if they had severe systemic medical illness, neurological disorders, history of head trauma with loss of consciousness, IQ<70 (assessed by the Wechsler Abbreviated Scale of Intelligence)[23], positive urine drug and/or salivary alcohol screen on the day of the scan, alcohol or substance abuse in the past three months (determined by The Schedule for Affective Disorders and Schizophrenia for School Age Children, Present and Life Version with WASH-U mood supplement; K-SADS-PL-W) [24], less than 20/40 Snellen visual acuity, using non-psychotropic medication with Central Nervous System (CNS) effects (i.e., steroids), being unable to complete questionnaires in English, history of physical/sexual abuse, autistic spectrum disorders or developmental delays. Additional exclusion criteria for scanning included pregnancy, claustrophobia, or metal objects in the body.

Because of data loss and excessive head movement (>4 mm) during scanning, data from 22 LAMS youth were excluded, leaving data on 85 LAMS youth. In addition, in order to define a more homogenous sample, which is important for the success of pattern recognition analysis (a common assumption of pattern recognition models is that the training examples are generated from the same distribution), we defined additional conservative exclusion criteria for the sample. This conservative exclusion criterion was employed to reduce, as much as possible, noise in the data which can particularly affect pattern recognition analyses. Firstly, we excluded subjects that had any evidence of acquisition artifact (10 subjects). Secondly, we limited the age range from 12–17 years, as this is a developmental period at which puberty is well underway in American youth [25,26]. Here, data from 18 LAMS youth were excluded as they were aged 11 or younger at the scanning session. A total of 57 LAMS2 youth (Age: M = 14.5, SD = 1.5, Range = 12–17, subsample of main LAMS2 sample) were thus included in the present study. Demographic information of the total LAMS2 (n = 107), main LAMS2 sample (n = 85) [12] and the participants used in this study were included on Table A in S1 Text.

The pediatric DSM-IV [25] primary and comorbid diagnoses were derived from the Schedule for Affective Disorders and Schizophrenia for School Age Children (K-SADS) [24] and appear in Table 1 (Table B in S1 Text presents demographic information, clinical variables, and current medication usage for the 57 youth from the three neuroimaging sites). A board-certified child psychiatrist or psychologist reviewed and confirmed the diagnoses. At the time of neuroimaging, 30 (52.6%) of participants were using at least one psychotropic medication. Of those, ten were taking two classes of psychotropic medications and two were taking three classes of psychotropic medications (Table 1). They were not asked to refrain from using prescribed medication(s) before and on the day of scanning.

**Table 1:** Demographic information, clinical variables, and current medication usage describing LAMS youth.

Descriptors	LAMS youth (N = 57)
	(M±SD or %)
**Age**	14.5 (1.5)
**IQ**	102.4 (17.4)
**SES (maternal education)**	
No/some HS	1.8
GED or HS Diploma	24.6
Some post HS	26.3
Associate’s Degree	28.1
Bachelor’s Degree or higher	19.3
**Sex (males)**	56.1
**Clinical Measures**	
PGBI-10M at screen	15.5 (6.3)
PGBI-10M near scan	4.7 (5.4)
**Current Diagnoses**	
ADHD	17.5
Anxiety Disorder	10.5
BPSD	36.8
Depressive Disorder	29.8
Disruptive Disorder	14.0
**Current Medication Use**	
Antidepressant	8.8
Antipsychotic	19.3
Benzodiazepine	0.0
Mood Stabilizer	5.3
Non-stimulant ADHD	7.0
Stimulant	36.8

Abbreviations: ADHD = Attention deficit hyperactivity disorder; BPSD = Bipolar Spectrum Disorder; HS = high school; IQ = intelligence quotient via Wechsler Intelligence test; PGBI-10M = Parent General Behavior Inventory 10 Item Mania scale; SES = socio-economic status–Maternal Education.

### Symptom Assessment

The PGBI-10M is a 10-item empirically-derived adaptation of the General Behavior Inventory [27,28]. Parents rate hypomanic, manic and biphasic mood symptoms of their children. Each item is scored from 0 (“Never or hardly ever”) to 3 (“very often or almost constantly”). Scores range from 0 to 30 with higher scores indicative of greater symptoms (please see S1 Text for PGBI-10M Assessment). The PGBI-10M was used to screen participants before entry into LAMS1 (at least five years before the neuroimaging scanning session in LAMS2). PGBI-10M scores were then obtained every six months in all LAMS youth throughout LASM1 and LAMS2. The PGBI-10M score at screen was included in analyses as the earliest measure of PGBI-10M score (*PGBI-10M at screen*, mean = 15.5, SD = 6.3, Range = 1–30). The PGBI-10M score nearest the scanning session (within six months) was included in analyses as a measure of most recent PGBI-10M score (*PGBI-10M close to the fMRI scanning session*, mean = 4.7, SD = 5.4, Range = 0–24).

### fMRI Paradigm

A block-design reward fMRI task was used comprising three experimental conditions “win”, “loss" and control blocks. At the beginning of each guessing trial, participants pressed a button to guess whether they thought the value of a visually presented card (with a possible value of one to nine, but whose value was not yet revealed) would be higher or lower than five (3000 msec). The actual numerical value of the card (500 msec), outcome feedback (Win: green upward-facing arrow; Loss: red downward-facing arrow, 500 msec) and a fixation cross (3000 ms) was then visually presented. Control trials required participants to press a button to the letter “X” (3000 msec) and view an asterisk (500 msec), yellow circle (500 msec), and fixation cross (3000 msec).

The fMRI paradigm comprised a total of nine blocks: three “win” blocks (80% win trials, 20% loss trials), three “loss" blocks (80% loss trials, 20% win trials), and three control blocks (no change in earnings). Five trials with an oddball format were presented in a predetermined outcome order in the guessing block (Win and Loss) (i.e. Win block: win, win, win, loss, win and Loss block: loss, loss, win, loss, loss). Participants completed the entire experimental paradigm in approximately six minutes. Participants were naive to the fixed outcomes, and believed outcomes to be randomly presented. The control blocks consisted of six trials. Before scanning, participants practiced the task and underwent scanner simulation to become familiar with the scanner environment and learn to avoid head movement before scanning. Participants were encouraged to perform to the best of their abilities and to minimize movement in the scanner. Participants were not debriefed about predetermined outcomes because they were to perform the task 18 months later.

### Image Acquisition

Neuroimaging data were collected using three different scanners: 1) 3T Siemens Verio MRI scanner at CWRU, 2) 3T Philips Achieva X-series MRI scanner at CCH, and 3) 3T Siemens Trio MRI scanner at UPMC [12]. Structural axial 3D Magnetization Prepared Rapid Gradient Echo (MP-RAGE) images were acquired with the following parameters: TE = 3.93 msec, TR = 2300 msec, flip angle = 9°; field of view = 256 x 192 mm, slice thickness = 1mm, image matrix = 256x192, 192 axial slices. Mean blood-oxygenation-level-dependent (BOLD) images were acquired with a reverse gradient-echo EPI sequence: 38 axial slices (3.1 mm thick, flip angle = 90°; field of view = 205 mm, TR = 2000 msec, TE = 28 msec, matrix = 64x64), encompassing the entire cerebrum and the majority of the cerebellum.

### Data Pre-processing and General Linear Model Analysis

Neuroimaging data were pre-processed using standard procedures in SPM8 (http://www.fil.ion.ucl.ac.uk/spm/) and involved realignment using the first slice as a reference, coregistration with the participant's MP-RAGE image, segmentation, normalization into a standard stereotactic space (Montreal Neurologic Institute, MNI; http://www.bic.mni.mcgill.ca), and spatially smoothing using a Gaussian kernel (FWHM: 8mm). For each participant, a General Linear Model (GLM) was implemented in SPM8 in which the effect of each condition (i.e. "win" and "loss") was modeled by the convolution of the blocks with the hemodynamic response function. Movement parameters from the realignment stage were entered in the GLM as nuisance covariates. Images corresponding to the GLM coefficients for each condition ("win" and "loss") defined the spatial patterns of brain activation used as input to the pattern regression analysis. To reduce inter-scan site variability, as recommended by the Biomedical Informatics Research Network (BIRN; standards detailed at http://www.nbirn.net), the signal:noise was monitored monthly using a BIRN phantom at all three sites to ensure scanner signal stability over time [12]. Finally a customized mask was created to include only brain voxels, which were common to all participants in the GLM coefficients (i.e. we excluded voxels which had a NaN (Not a Number) in the GLM coefficient for at least one participant). We recently noticed that this criterion for creating the mask can significantly improve the performance of pattern recognition analyses by decreasing the number of non informative features/voxels in the model. Please see on Table D in S1 Text the results obtained when we used the standard (whole-brain) template from the Pattern Recognition for Neuroimaging Toolbox (PRoNTo, http://www.mlnl.cs.ucl.ac.uk/pronto/) as the first level mask.

### Pattern Regression Analysis

**Fig 1:**
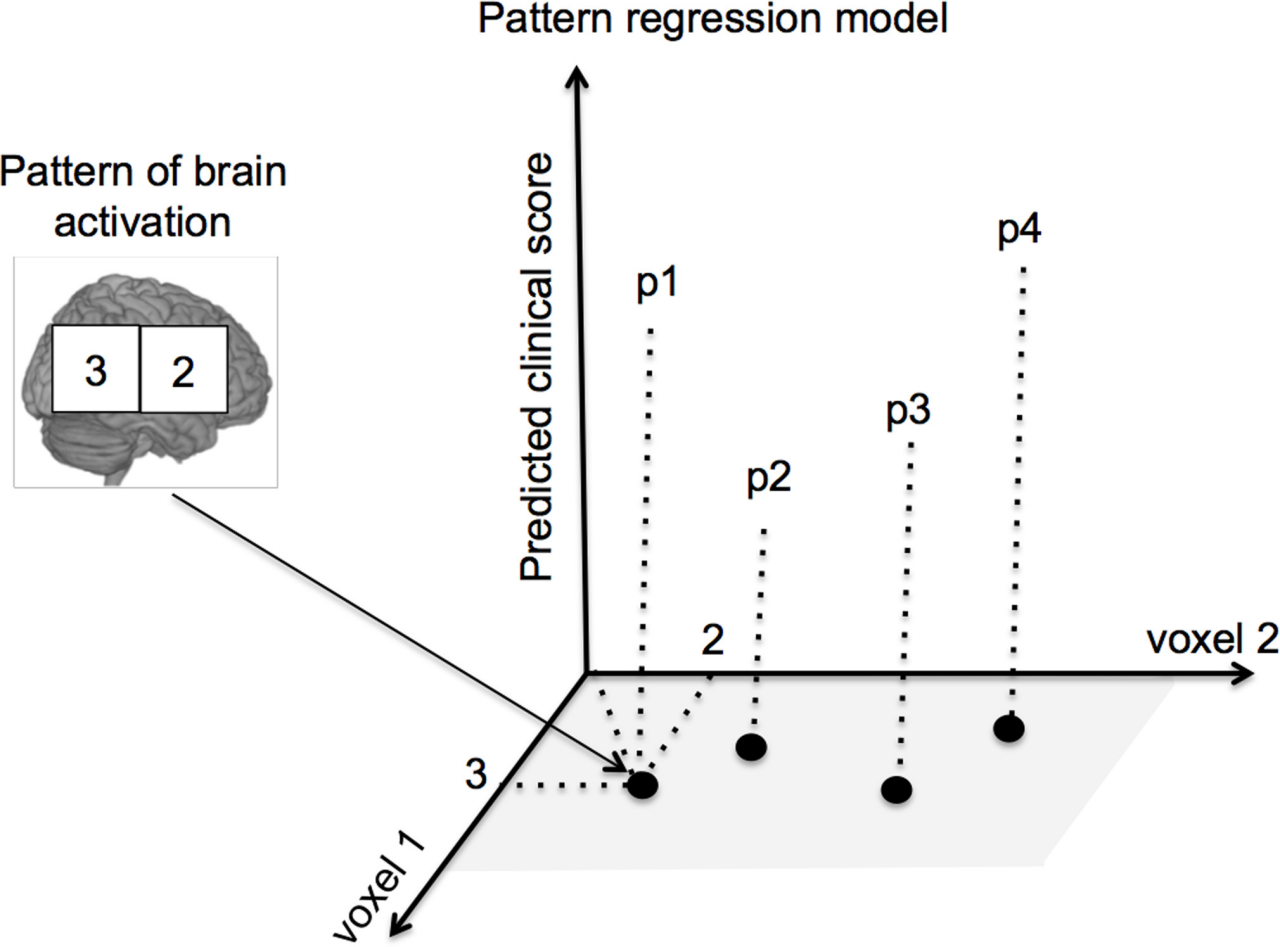
Illustration of a pattern regression model with hypothetical 2D data. The numbers in the squares correspond to activation levels in different brain voxels. Each brain scan corresponds to a point in a 2D voxel space. The goal is to “train” a predictive function that given a new brain scan can accurately decode the correspondent clinical score.

The procedure for building pattern regression models consists of two phases: training and testing. During the training phase, the model is trained by providing examples that pair a spatial pattern (e.g., pattern of brain activity during an experimental condition) and a label (e.g., clinical score). Once the model has “learned” the association between patterns and labels from the training data, it can be used to decode or predict the label of a new test example (Fig 1). We used the standard procedure in PRoNTo to train and test the model. The data set is partitioned into training and testing sets (the number of subjects in each set depends on the cross-validation scheme chosen as explained in the next section). The model is then trained to learn the association between the patterns of brain activity and the PGBI-10M score using the training set examples. After that the model is tested, i.e. given the patterns of brain activation of the test subjects the model predicts their scores. Finally the actual and predicted PGBI-10M scores are compared using different metrics (as described later). This procedure is repeated a number of times according to the cross-validation scheme chosen and the results are averaged across these repetitions. We trained one model for each condition (win and loss) and for each time point of the PGBI-10M scores (at screen and closest to scan). In addition we applied the operation to mean centre the features using the training data. The choice of machine learning algorithm depends on many factors, such as generalization performance measured on test data and computational cost of the algorithm. In preliminary investigations we compared the performance of three different algorithms currently available in PRoNTo [29]: Relevance Vector Regression (RVR) [30], Gaussian Process Regression (GPR) [31] and Kernel Ridge Regression (KRR) [32]. There were no significant differences in performance for the three different approaches. For the sake of brevity we present results only for RVR. RVR is a sparse kernel-based machine learning algorithm formulated in a Bayesian framework, and has been previously applied to neuroimaging based predictions [16,30,33].

RVR was applied to decode PGBI-10M scores recorded at two time points (*PGBI-10M at screen* and *PGBI-10M closest to scan*) from patterns of brain activity during win and loss blocks of the reward task (i.e. we trained one independent RVR model for each time point). These two clinical scores are referred to as “labels”.

### Cross-Validation Strategies

To evaluate the RVR performance, we used two different *cross-validation* strategies: leave-one-out cross-validation and four-fold cross-validation. The leave-one-out cross-validation is a frequently used validation method and involves leaving one participant out for test and train the model on *N*-1 participants (doing so *N* times so that each participant is left out once). The four-fold cross-validation involves dividing the data into 4 disjoint folds. Data from each fold is left out once for test and data from the remaining 3 folds are used to train the model. This procedure is then repeated four times, so that each fold is left out once. Because our sample size was not divisible by four, three folds contained 14 participants each, while the remaining fold contained 15 participants. In the four-fold cross-validation procedure, we applied a t-test to insure that the distribution of the to-be-predicted variables (PGBI-10M *at screen* and *closest to scan*) did not differ among the folds. In both cross-validation strategies the clinical score of the participant(s) left out for test was/were decoded from the corresponding GLM coefficient image(s) using the RVR model trained on the remaining participants. Note that the leave-one-out procedure can also be considered a *N*-fold cross-validation where *N* is the number of subjects (in this case each fold contains a single subject).

We measured agreement between decoded and actual clinical scores using two different metrics: Pearson’s correlation coefficient (r) and mean squared error (MSE). The correlation coefficient describes the strength of a linear relationship between two variables. The higher this coefficient, the more accurate were the predictions. We also computed the MSE, which is the mean of the squared differences between the decoded and true scores. MSE represents the mean error between the decoded and actual clinical scores and is commonly used to evaluate the performance of predictive models. For these metrics, statistical significance was determined by permutation tests, i.e., we performed the same cross-validation procedures described above but with the labels permuted across participants. We repeated this procedure 1000 times and counted how many times the absolute value of the metric (r or MSE) with the permuted labels was equal to or higher than (or lower than in the case of the MSE) than the one obtained with the correct labels. The *p*-value was then calculated by dividing this number by the number of permutations (1000). Results were considered significant if the *p*-value< 0.05.

### Medication

Medication was considered as a potential confounder, and was regressed out of the pattern regression analysis using the residual forming matrix framework [33–35]. Medication was treated as a binary variable, i.e. 1 for medicated participants and 0 for non-medicated participants. This procedure is equivalent to dummy variable regression or analysis of covariance (ANCOVA) which is frequently used in hope of “controlling for” group differences using covariates. It should be emphasized that applying ANCOVA to control for covariates not randomly sampled across groups is statistically inappropriate, given that if the variable to be predicted or decoded is associated with the confound variable, no statistical approach can remove the effect of this confound or covariate [36]. In the present study, we used two sample t-tests to determine whether medication was systematically related to the clinical scores that we intended to decode.

### Pattern Localization

For pattern regression models showing significant values of correlation and MSE, weight maps were built. For linear models, the weight map is a voxel space representation of the model’s parameters or decision function. Because each cross-validation fold yields a different weight vector, the final weight map is the average map across folds divided by its Euclidean norm. Linear pattern recognition models generate weights for each voxel, while model predictions are based on the whole pattern. Thus, the weight map cannot be thresholded to make regionally-specific inferences as in classical (univariate) techniques. Here we applied a novel methodology, referred to as pattern localization, based on a labelled anatomical template to summarize the weight map in terms of anatomical regions [37]. Briefly, for each brain region defined by the anatomical template, we computed the mean of absolute values of all voxel weights within this region (normalized by the number of voxels). We then ranked the labelled regions according to the percentage of the total normalized weights they explained. We used the Anatomical Automatic Labeling (AAL) atlas from the WFU-PickAtlas [38] toolbox in SPM to define the regions.

## Results

### Effect of Medication on Clinical Scores

In the present study, we used two sample t-tests to determine whether medication was systematically related to the clinical scores that we intended to decode. Youth taking medication did not score significantly differently than youth not taking medication on PGBI-10M at screen (*p*-value = 0.70, medicated: M = 15.9, SD = 5.8, unmedicated: M = 15, SD = 7.0) and on PGBI-10M closest to scan (*p*-value = 0.09, medicated: M = 5.8, SD = 6.3, unmedicated: M = 3.5, SD = 3.8).

### Pattern Regression

Model performance measuring agreement between decoded and actual labels for all models and cross-validation frameworks are presented in Table 2.

**Table 2:** Measures of agreement between actual and decoded clinical scores for PGBI-10M at screen and closest to scan, based on functional neuroimaging scans (win and loss blocks).

	Controlled for medication		Not-controlled for medication	
Measures	r (*p*-value)	MSE (p-value)	r (*p*-value)	MSE (*p*-value)
**Leave-one-out cross-validation (Win blocks)**				
**PGBI-10M at screen**	**0.43 (0.001)**	**32.35 (0.001)**	**0.40 (0.009)**	**33.68 (0.006)**
**PGBI-10M closest to scan**	**0.15 (0.14)**	**29.54 (0.07)**	**0.35 (0.01)**	**25.25 (0.009)**
**Four-fold cross-validation (Win blocks)**				
**PGBI-10M at screen**	**0.41 (0.001)**	**34.38 (0.001)**	**0.36 (0.01)**	**34.48 (0.007)**
**PGBI-10M closest to scan**	**0.02(0.40)**	**43.10 (0.69)**	**0.34 (0.01)**	**25.73 (0.008)**
**Leave-one-out cross-validation (Loss blocks)**				
**PGBI-10M at screen**	**0.46 (0.001)**	**31.12 (0.001)**	**0.38 (0.006)**	**34.53 (0.005)**
**PGBI-10M closest to scan**	**0.07 (0.24)**	**31.51 (0.12)**	**0.28 (0.03)**	**26.96 (0.02)**
**Four-fold cross-validation (Loss blocks)**				
**PGBI-10M at screen**	**0.34 (0.01)**	**38.95 (0.02)**	**0.30 (0.02)**	**37.88 (0.02)**
**PGBI-10M closest to scan**	**-0.01(0.48)**	**46.38 (0.76)**	**0.29 (0.03)**	**26.97 (0.02)**

Abbreviations: PGBI-10M = Parent General Behavior Inventory 10 Item Mania scale; r = Pearson’s correlation value, MSE = mean squared error.

### PGBI-10M at screen

#### Win blocks: Leave-one-out cross-validation framework

When controlling for medication effects, the correlation coefficient (r) and MSE between the decoded and actual PGBI-10M *at screen* were 0.43 (*p*-value = 0.001) and 32.35 (*p*-value = 0.001), respectively (Table 2). Similar results were obtained for PGBI-10M *at screen* without controlling for medication, indicating that our model was able to decode PGBI-10M from changes in brain activation due to the task, and not due to the effect of medication.

#### Win blocks: Four-fold cross-validation framework

When controlling for medication effects, the correlation coefficient (r) and MSE between the decoded and actual PGBI-10M *at screen* were 0.41 (*p*-value = 0.001) and 34.38 (*p*-value = 0.001), respectively. Similar significant results were obtained without controlling for potential medication effects (Table 2).

#### Loss blocks: Leave-one-out cross-validation framework

When controlling for medication effects, the correlation coefficient (r) and MSE between the decoded and actual PGBI-10M *at screen* were 0.46 (*p*-value = 0.001) and 31.12 (*p*-value = 0.001), respectively. Similar significant results were obtained without controlling for potential medication effects (Table 2).

#### Loss blocks: Four-fold cross-validation framework

When controlling for medication effects, the correlation coefficient (r) and MSE between the decoded and actual PGBI-10M *at screen* were 0.34 (*p*-value = 0.01) and 38.95 (*p*-value = 0.02), respectively. Similar significant results were obtained without controlling for potential medication effects (Table 2).

### PGBI-10M closest to scan

For both cross-validation frameworks (leave-one-out and four-folds cross-validation), the correlation coefficient and the MSE between actual and decoded symptoms were significant only for the models not controlling for medication effects (Table 2). After controlling for medication effects, the models were not able to predict PGBI-10M closest to scan (Table 2).

### Voxel-Based Predictive Patterns

**Fig 2:**
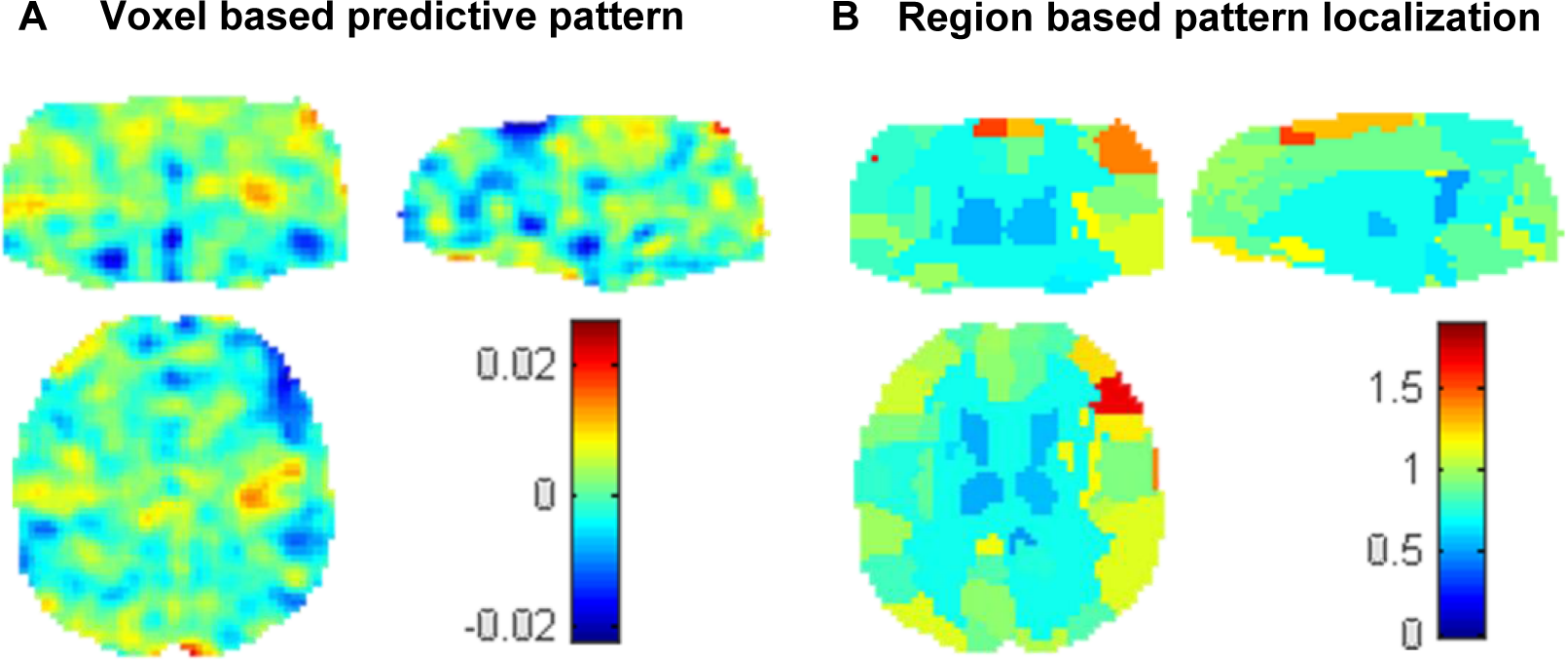
Maps for decoding PGBI-10M at screen based on patterns of activation to win blocks using a leave-one-out cross-validation framework. A: Voxel-based predictive pattern. The colour bar indicates the weight of the voxels for decoding the clinical score. B: Region-based pattern localization map computed from the voxel based predictive pattern displayed in Fig 2A. The colour bar indicates the percentage of the total normalized weights that each anatomically labelled region explains.

Due to space limitation, and to avoid large number of tables in the main manuscript, we choose to display only the models using the leave-one-out cross-validation based on the win blocks which were significant after controlling for medication effects. Please see on Table C in S1 Text and Figs Aa and Ab in S1 Text the results using the leave-one-out cross-validation based on the loss blocks. In Fig 2A we present the spatial pattern that decoded PGBI-10M at screen based on patterns of brain activation to win blocks using the leave-one-out cross-validation procedure. The weight in each voxel corresponds to its contribution to the model’s prediction. We emphasize that weight maps should not be interpreted as statistical parametric maps; they simply provide a spatial representation of the decision function and should not be thresholded, as all voxels within the mask contributed to the final predictions.

### Pattern Localization

Fig 2B depicts the region-based pattern localization map (computed from the voxel based predictive pattern displayed in Fig 2A). The colour of each region corresponds to the normalized average of voxels weights within the regions (in absolute value). Table 3 displays the top 20 ranked regions according to normalized weights per region, which represent 29.6% of the total weights in the decision function (for completeness we included Table E in S1 Text and Table F in S1 Text with the complete list of ranked regions). The regions with highest contributions to the predictions were fronto- tempo-parietal regions, motor areas, insula and the cerebellum. Contributions of individual regions were very small, however, suggesting that predictions were based on the overall pattern rather than on a small combination of regions.

**Table 3:** The top 20 ranked brain regions according to the normalized weights (NW) to decode PGBI-10M at screen based on pattern of brain activation to win blocks using a leave-one-out cross-validation framework. The listed regions represent 29.6% of the total weights in the decision function.

Rank	Brain Regions	% NW(ROI)
1	Frontal Inf Oper L	1.9
2	Cerebelum 4 5 L	1.7
3	Precuneus L	1.7
4	Parietal Inf R	1.7
5	Parietal Sup R	1.7
6	Frontal Inf Orb L	1.7
7	Parietal Inf L	1.6
8	Cerebelum 3 R	1.6
9	Insula L	1.6
10	Supp Motor Area R	1.5
11	Calcarine L	1.4
12	Temporal Pole Sup R	1.4
13	Parietal Sup L	1.4
14	Rectus R	1.3
15	Frontal Mid Orb R	1.3
16	Olfactory L	1.3
17	Fusiform L	1.2
18	Cerebelum Crus1 L	1.2
19	Frontal Mid Orb L	1.2
20	SupraMarginal L	1.2

Abbreviations: Inf: Inferior; L: Left; Med: Medial; Mid: Middle; Oper: Opercularis, Orb: Orbital; Post: Posterior; R: Right; Sup: Superior; Supp: Supplementary; % NW(ROI): Percentage of the total normalized weights that each anatomical region explains.

## Discussion

The main goal of the present study was to apply pattern regression analysis to functional neuroimaging to determine whether a dimensional scale measuring behavioural and emotional dysregulation at different time points (initial study screen and closest to the fMRI scanning section) could be decoded from patterns of whole-brain activity in a large multi-center of youth with a variety of different diagnoses, including BPSD, other mood disorders, ADHD, anxiety, and disruptive disorders. Our results show that pattern recognition models have the potential to decode individual-level severity on a dimensional scale measuring behavioural and emotional dysregulation from individual-level patterns of brain activity. These results represent early evidence that neuroimaging techniques may inform the clinical assessment of psychiatric youth by allowing accurate and objective quantitative estimation of psychopathology.

The functional neuroimaging data used in the current study have been analyzed and reported previously by Bebko *et al* 2014[12]. There, the authors applied voxel-wise statistical analyses to the whole LAMS2 sample (85 subjects) with the aim of identifying local measures of activity in reward processing neural circuitry related to pathological dimensions. They showed significant positive associations between activity in the left middle prefrontal cortex and PGBI-10M scores nearest the scanning session, and between activity in the right dorsal anterior cingulate cortex and anxiety scores. As in [12], the vast majority of work in the literature has focused on finding associations between signal of individual regions and proximal measures [12,13,39]. Our work differs from Bebko *et al* 2014 as, instead of investigating association between symptoms and brain activity in individual brain regions at the group level using conventional neuroimaging data analytic techniques (univariate analysis), we applied machine learning techniques, with the aim of determining whether measures of behavioural and emotional dysregulation at different time points (operationalized as PGBI-10M scores) could be decoded from patterns of whole-brain activity. To the best of our knowledge, there is only one previous study applying pattern regression to predict individual proximal clinical assessment in adults psychiatric patients from patterns of brain activity using fMRI [40].

Our findings show that pattern regression analysis has the potential to decode behavioral and emotional dysregulation symptoms from patterns of brain activation during a reward task in individual LAMS2 youth not only close to the scanning session, but also at the initial study screen. The correlation and mean squared error between actual and decoded measures were significant when the model was based on patterns of brain activation to the *win blocks* and to *the loss blocks* and also when using different cross-validation strategies (leave-one-out and four-fold cross-validation). The initial screen measures of behavioral and emotional dysregulation were obtained when youth entered LAMS1 several years prior to the neuroimaging scan in LAMS2 (at least 5 years earlier). These results suggest that the relationship between PGBI-10M score and the brain activity is stable over the years.

An unexpected aspect of our findings is that the correlation value (r) between the predicted and actual PGBI-10M was higher when the model predicted PGBI-10M *at screen* than *closest to scan*. In addition, when controlling for medication the models were not able to predict PGBI-10M *closest to scan*. One possible explanation for these counterintuitive findings is the fact that the PGBI-10M score decreased for both medicated and non-medicated youth relative to PGBI-10M scores obtained at the study screen, such that the distribution of the PGBI-10M scores closest to the scanning session was skewed towards low values. This could have made it more difficult for the learning algorithm to learn the relationship between the full range of PGBI-10M scores and brain activity. Furthermore, the procedure applied to control for potential medication effects removed the variability in the data (patterns of brain activity) associated with medication. Although this approach is commonly used when controlling for nuisance variables, it can potentially remove information from the data which is useful for prediction [41]. In the present study, youth taking medication scored slightly differently than youth not taking medication on PGBI-10M *closest to scan*, as suggested by the lower p-value (0.09). It is possible that by removing the variability from the data associated with medication we may have also removed important predictive information for PGBI-10M *closest to scan*.

Interestingly, the brain regions with the highest contribution to decoding the PGBI-10M score at initial study screen were fronto-parietal regions, sensory-motor areas, insula and cerebellum. These regions include many neural regions important for emotional regulation [42,43], attention [44], and novelty and salience perception [45–47]. Interestingly, the region with the highest contribution to decoding PGBI-10M score at initial study screen was the left opercular region, part of the left ventrolateral prefrontal cortex, greater activity within which has previously been associated with heightened reward sensitivity and thrill-seeking, and with bipolar disorder [43]. It is well established that psychiatric disorders, such as anxiety, depression, bipolar and attention deficit/hyperactivity disorder, are associated with abnormal functioning within these regions [43,48–50]. As a result, these findings suggest that patterns of neural activity in regions important for emotional regulation and salience perception in the context of receiving monetary reward or loss may contribute to behavioral and emotional dysregulation in youth. Our investigation therefore indicates that these neural regions may provide critical information for successfully estimating emotional and behavioural symptomatology at the individual level. As highlighted above, however, these findings should be interpreted with caution, as the predictive spatial pattern provides a spatial representation of the decision function, with all voxels within the mask contributing to the final prediction.

A strength of the current study was the inclusion of two different cross-validation strategies. Although it is common to leave one participant out and train the model with *N*-1 participants (where *N* corresponds to the total number of participants), demonstration of reproducibility between different cross-validation strategies is important to show the stability of the results and for future clinical use of machine learning methods. In addition, one of the biggest challenges for the translation of neuroimaging-based predictive models into clinical practice is the validation of models using large multi-center datasets. This validation is important to demonstrate the robustness of models with regard to variability caused by scanner type and acquisition protocols. Given that participants were scanned at multiple sites, our findings demonstrate that the pattern regression model was able to treat the site effect as noise and learn a common predictive pattern across different sites, which highlights the robustness of the model when applied to data from different scanners.

There were some limitations in the present study. One important issue is the potential confounding effect of medication on neuroimaging data. Most participants of this study were medicated (n = 30), and of those, 12 were taking more than one class of psychotropic medications. As explained in the methods and results sections, to address this issue medication was treated as a binary variable (medicated versus non medicated) and regressed out of the pattern regression analysis using the residual forming matrix framework [34,35] when decoding PGBI-10M at the initial study screen and closest to the scanning session. Considering the potentially complex effect of different medications and their interactions on brain activation, treating medication as a binary variable is a simplistic approach. Nevertheless this is a very common limitation in studies with realistic patients given the absence of more precise models of the combined influence of different CNS drugs on brain activation. Second, even though we applied two different cross-validation strategies to demonstrate generalizability of the model, ideally the model should be trained and tested with truly independent samples. Further studies with larger sample sizes are needed to assess the generalizability of the proposed approach by training and testing with completely independent samples. Finally, we are aware that Pearson’s correlation coefficients on their own do not provide a satisfactory way of evaluating the model's performance. For this reason, we only considered the predictive models to be significant when the correlation coefficient and the mean squared error's p-values (estimated using permutation tests) were both below the significance threshold of 0.05.

The present study was a proof of concept study designed to examine whether pattern regression analysis could be applied to neuroimaging to decode individual-level severity along dimensions of behavioural measures. Our results suggest that it is possible to identify patterns of neural activity associated with symptoms of behavioral and emotional dysregulation from whole-brain patterns of activity during a reward-processing task in youth. Future studies, using a combination of pattern regression and neuroimaging, can build on the present findings to determine the extent to which individual-level patterns of neural function either instead of or in combination with clinical, familial and demographic measures can predict individual-level future clinical outcomes. Furthermore multivariate predictive models such as the one used in the current study could prove useful for predicting the effect of a given treatment.

## Supporting Information

S1 Text Additional text, list of Tables A-F and figure including detailed demographic information and clinical variables describing the LAMS participants along with additional results. Table A: Demographic information, clinical variables, and current medication usage (Mean ± Standard Deviation or Proportion) describing the LAMS participants (Total LAMS, Main LAMS2 (Bebko’s paper[5]), Included Participants). Table B: Demographic information, clinical variables, and current medication usage (Mean ± Standard Deviation or Proportion) describing the 57 youth LAMS from the three neuroimaging sites. Table C: The top 20 ranked brain regions according to the normalized weights (NW) to decode PGBI-10M at screen based on pattern of brain activation to loss blocks using a leave-one-out cross-validation framework. Table D: Measures of agreement between actual and decoded clinical scores for PGBI-10M at screen and closest to scan, based on functional neuroimaging scans (win and loss blocks) using the standard (whole-brain) template from the Pattern Recognition for Neuroimaging Toolbox (PRoNTo, http://www.mlnl.cs.ucl.ac.uk/pronto/). Table E: The complete list of brain regions according to the normalized weights (NW) to decode PGBI-10M at screen based on pattern of brain activation to win blocks using a leave-one-out cross-validation framework. Table F: The complete list of brain regions according to the normalized weights (NW) to decode PGBI-10M at screen based on pattern of brain activation to loss blocks using a leave-one-out cross-validation framework. Fig A: Maps for decoding PGBI-10M at screen based on patterns of activation to loss blocks using a leave-one-out cross-validation framework. Aa: Voxel-based predictive pattern. The colour bar indicates the weight of the voxels for decoding the clinical score. Ab: Region-based pattern localization map computed from the voxel based predictive pattern displayed in Fig Aa. The colour bar indicates the percentage of the total normalized weights that each anatomically labelled region explains.
(DOC)
