# Supplementary material for: Can Emotional and Behavioral Dysregulation in Youth Be Decoded from Functional Neuroimaging?
Source: PLoS One. 2016 Jan 5;11(1):e0117603. doi: 10.1371/journal.pone.0117603 (PMC4701457; doi:10.1371/journal.pone.0117603)
Supplement: S1 Text — Table A: Demographic information, clinical variables, and current medication usage (Mean ± Standard Deviation or Proportion) describing the LAMS participants (Total LAMS, Main LAMS2 (Bebko’s paper[5]), Included Participants). Table B: Demographic information, clinical variables, and current medication usage (Mean ± Standard Deviation or Proportion) describing the 57 youth LAMS from the three neuroimaging sites. Table C: The top 20 ranked brain regions according to the normalized weights (NW) to decode PGBI-10M at screen based on pattern of brain activation to loss blocks using a leave-one-out cross-validation framework. Table D: Measures of agreement between actual and decoded clinical scores for PGBI-10M at screen and closest to scan, based on functional neuroimaging scans (win and loss blocks) using the standard (whole-brain) template from the Pattern Recognition for Neuroimaging Toolbox (PRoNTo, http://www.mlnl.cs.ucl.ac.uk/pronto/). Table E: The complete list of brain regions according to the normalized weights (NW) to decode PGBI-10M at screen based on pattern of brain activation to win blocks using a leave-one-out cross-validation framework. Table F: The complete list of brain regions according to the normalized weights (NW) to decode PGBI-10M at screen based on pattern of brain activation to loss blocks using a leave-one-out cross-validation framework. Fig A: Maps for decoding PGBI-10M at screen based on patterns of activation to loss blocks using a leave-one-out cross-validation framework. Aa: Voxel-based predictive pattern. The colour bar indicates the weight of the voxels for decoding the clinical score. Ab: Region-based pattern localization map computed from the voxel based predictive pattern displayed in Fig Aa. The colour bar indicates the percentage of the total normalized weights that each anatomically labelled region explains. (DOC) [file pone.0117603.s001.doc]

**S1 Supporting Information**

**Titles for S1 Text:**

Table A: Demographic information, clinical variables, and current medication usage (Mean ± Standard Deviation or Proportion) describing the LAMS participants (Total LAMS, Main LAMS2 (Bebko’s paper[5]), Included Participants).

Table B: Demographic information, clinical variables, and current medication usage (Mean ± Standard Deviation or Proportion) describing the 57 youth LAMS from the three neuroimaging sites.

Table C: The top 20 ranked brain regions according to the normalized weights (NW) to decode PGBI-10M at screen based on pattern of brain activation to loss blocks using a leave-one-out cross-validation framework.

Table D: Measures of agreement between actual and decoded clinical scores for PGBI-10M at screen and closest to scan, based on functional neuroimaging scans (win and loss blocks) using the standard (whole-brain) template from the Pattern Recognition for Neuroimaging Toolbox (PRoNTo, <http://www.mlnl.cs.ucl.ac.uk/pronto/>).

Table E: The complete list of brain regions according to the normalized weights (NW) to decode PGBI-10M at screen based on pattern of brain activation to win blocks using a leave-one-out cross-validation framework.

Table F: The complete list of brain regions according to the normalized weights (NW) to decode PGBI-10M at screen based on pattern of brain activation to loss blocks using a leave-one-out cross-validation framework.

Figure A: Maps for decoding PGBI-10M at screen based on patterns of activation to loss blocks using a leave-one-out cross-validation framework. Aa: Voxel-based predictive pattern. The colour bar indicates the weight of the voxels for decoding the clinical score. Ab: Region-based pattern localization map computed from the voxel based predictive pattern displayed in Figure Aa. The colour bar indicates the percentage of the total normalized weights that each anatomically labelled region explains.

**Description of LAMS**

LAMS is a longitudinal NIMH-supported study of 6-12 year old children recruited at their first visit to nine mental health clinics associated with four universities [1]. Children were screened with the Parent General Behavior Inventory-10 Item Mania Scale (PGBI-10M). All children scoring >=12 on the PGBI-10M, and a demographically matched sample of children who scored <=11 who were also seeking mental health care but did not have severe behavioral or emotion dysregulation, were invited to participate. For a more detailed account of the LAMS background and study design, please refer to Horwitz and colleagues [2].

**Diagnostic Criteria**

We used unmodified DSM-IV diagnostic criteria. We defined bipolar spectrum disorders (BPSD) as a diagnosis of bipolar I disorder; bipolar I disorder single episode; bipolar I disorder most recent episode hypomanic; bipolar I disorder most recent episode manic; bipolar I disorder most recent episode mixed; bipolar I disorder most recent episode depressed; bipolar I disorder most recent episode unspecified; bipolar II disorder; bipolar II disorder most recent episode hypomanic; bipolar II disorder most recent episode depressed; subsyndromal bipolar disorder; cyclothymic disorder; or bipolar disorder not otherwise specified. We defined disruptive disorders as a diagnosis of conduct disorder, disruptive disorders not otherwise specified, and oppositional defiant disorder. A board-certified child psychiatrist reviewed and confirmed diagnoses.

**PGBI-10M Assessment**

The questions/items from the PGBI-10M [3,4] describe positive mood dysregulation and discriminate between BPSD and other comorbidities, such as ADHD. The 10 questions/items are listed below:

*1. Has your child experienced periods of several days or more when, although he/she was feeling unusually happy and intensely energetic (clearly more than your child’s usual self), he/she was also physically restless, unable to sit still, and had to keep moving or jumping from one activity to another?*

*2. Have there been periods of several days or more when your child’s friends or other family members told you that your child seemed unusually happy or high – clearly different from his/her usual self or from a typical good mood?*

*3. Has your child’s mood or energy shifted rapidly back and forth from happy to sad or high to low?*

*4. Has your child had periods of extreme happiness and intense energy lasting several days or more when he/she also felt much more anxious or tense (jittery, nervous, uptight) than usual (other than related to the menstrual cycle)?*

*5. Have there been times of several days or more when, although your child was feeling unusually happy and intensely energetic (clearly more than his/her usual self), he/she also had to struggle very hard to control inner feelings of rage or an urge to smash or destroy things?*

*6. Has your child had periods of extreme happiness and intense energy (clearly more than his/her usual self) when, for several days or more, it took him/her over and hour to get to sleep at night?*

*7. Have you found that your child’s feelings or energy are generally up or down, but rarely in the middle?*

*8. Has your child had periods lasting several days or more when he/she felt depressed or irritable, and then other periods of several days or more when he/she felt extremely high, elated, and overflowing with energy?*

*9. Have there been periods when, although your child was feeling unusually happy and intensely energetic, almost everything got on his/her nerves and made him/her irritable or angry (other than related to the menstrual cycle)?*

*10. Has your child had times when his/her thoughts and ideas came so fast that he/she couldn’t get them all out, or they came so quickly others complained that they couldn’t keep up with your child’s ideas?*

Table A: Demographic information, clinical variables, and current medication usage (Mean ± Standard Deviation or Proportion) describing the LAMS participants (Total LAMS, Main LAMS2 (Bebko’s paper[5]), Included Participants).

|  | | Total LAMS 2 Sample  N=107 | | Main LAMS2 | Included Participants  N=57 | |
| --- | --- | --- | --- | --- | --- | --- |
|  | |  |  | N=85 |  |  |
| **Demographic Information** | | | |  |  | |
| Age | | 13.4(2.0) | | 13.7(2.0) | 14.4(1.5) | |
| IQ | | 100.2(16.5) | | 102.4(16.9) | 102.4(17.4) | |
| SES | |  |  |  |  | |
|  | No/some HS | 7/107 | | 4/85 | 1/57 | |
|  | GED or HS Diploma | 25/107 | | 21/85 | 14/57 | |
|  | Some post HS | 24/107 | | 18/85 | 15/57 | |
|  | Associate’s Degree | 30/107 | | 25/85 | 16/57 | |
|  | Bachelor’s Degree or higher | 21/107 | | 17/85 | 11/57 | |
| Sex (females) | | 45/107 | | 39/85 | 25/57 | |
| **Clinical Measures** | |  | |  |  | |
| PGBI-10M screen | | 16.0(6.2) | | 16.0 (6.3) | 15.5(6. 3) | |
| PGBI-10M near scan | | 6. 1(5.8) | | 6.1(5.7) | 4.7(5.4) | |
| **Current Medication Use** | | | |  |  |  |
| Antidepressant | | 18/107 | | 14/85 | 5/57 | |
| Antipsychotic | | 26/107 | | 23/85 | 11/57 | |
| Benzodiazepine | | 1/107 | | 0/85 | 0/57 | |
| Mood Stabilizer | | 8/107 | | 6/85 | 3/57 | |
| Non-stimulant | | 9/107 | | 6/85 | 4/57 | |
| Stimulant | | 42/107 | | 36/85 | 21/57 | |

Abbreviations: HS=high school; IQ=intelligence quotient Wechsler Intelligence test; K-DRS=Kiddie Schedule for Affective Disorders and Schizophrenia for School-Age Children Present Episode Depression Rating Scale; K-MRS=Kiddie Schedule for Affective Disorders and Schizophrenia for School-Age Children Mania Rating Scale; PGBI-10M=Parent General Behavior Inventory 10 Item Mania scale; SCARED=Screen for Child Anxiety Related Emotional Disorders (child rating); SES=socio-economic status–Maternal Education.

Table B: Demographic information, clinical variables, and current medication usage (Mean ± Standard Deviation or Proportion) describing the 57 youth LAMS from the three neuroimaging sites.

|  | |  | Cleveland | | Cincinnati | Pittsburgh | Test Statistic (df) | | *p* |  |
| --- | --- | --- | --- | --- | --- | --- | --- | --- | --- | --- |
| **Sample Size** | | | 18 | | 16 | 23 |  | |  |  |
| **Demographic Information** | | | | | | | | | |  |
| Age | | | 14.8 (1.7) | | 14.4 (1.3) | 14.3 (1.6) | F(2,54)= 0.6 | | 0.53 |  |
| IQ | | | 104.1(21.3) | | 105.0 (14.9) | 99.2 (16.2) | F(2,54)= 0.6 | | 0.53 |  |
| Sex (females) | | | 11/18 | | 4/16 | 10/23 | χ^2^(2)=4.48 | | 0.11 |  |
| **Clinical Measures** | | | | | | | | | |  |
| PGBI-10M at screen | | | 14.5 (8.1) | | 14.1 (5.7) | 17.30 (4.9) | F(2,54)= 1.5 | 0.23 | |  |
| PGBI-10M near scan | | | 4.9 (5.7) | | 3.8 (6.0) | 5.3 (4.8) | F(2,54)= 0.4 | 0.69 | |  |
| **Current Medication Use** | | | | | | | | | |  |
|  | Antidepressant | | | 1/18 | 2/16 | 2/23 | χ^2^(2)=0.51 | 0.77 | | |
|  | Antipsychotic | | | 1/18 | 5/16 | 5/23 | χ^2^(2)=3.73 | 0.15 | | |
|  | Benzodiazepine | | | 0/18 | 0/16 | 0/23 | --- |  | | |
|  | Mood Stabilizer | | | 1/18 | 0/16 | 2/23 | χ^2^(2)=1.44 | 0.49 | | |
|  | Non-stimulant | | | 2/18 | 1/16 | 1/23 | χ^2^(2)=0.72 | 0.69 | | |
|  | Stimulant | | | 3/18 | 8/16 | 10/23 | χ^2^(2)=4.77 | 0.09 | | |

Abbreviations: *=significant at *p*=.05; df=degrees of freedom; F=ANOVA test statistical value; IQ=intelligence quotient Wechsler Intelligence test; *p*=*p* value; PGBI-10M=Parent General Behavior Inventory 10 Item Mania Scale; χ^2^=chi-squared test statistic value.

Table C: The top 20 ranked brain regions according to the normalized weights (NW) to decode PGBI-10M at screen based on pattern of brain activation to loss blocks using a leave-one-out cross-validation framework.

| **Rank** | **Brain Regions** | **% NW(ROI)** |
| --- | --- | --- |
| 1 | Cerebelum 4 5 L | 2.2 |
| 2 | Frontal Inf Oper L | 2.1 |
| 3 | Parietal Inf L | 2.0 |
| 4 | Cerebelum 3 R | 1.6 |
| 5 | Supp Motor Area R | 1.6 |
| 6 | Parietal Sup R | 1.6 |
| 7 | Frontal Mid Orb R | 1.5 |
| 8 | Frontal Inf Orb L | 1.5 |
| 9 | Cingulum Post R | 1.4 |
| 10 | Parietal Inf R | 1.4 |
| 11 | Temporal Sup L | 1.3 |
| 12 | Frontal Mid Orb R | 1.3 |
| 13 | Cerebelum 6 L | 1.3 |
| 14 | Rectus L | 1.3 |
| 15 | Frontal Inf Tri L | 1.3 |
| 16 | Parietal Sup L | 1.3 |
| 17 | Precuneus L | 1.3 |
| 18 | Cerebelum 4 5 R | 1.3 |
| 19 | Temporal Pole Sup R | 1.2 |
| 20 | Olfactory R | 1.2 |

The listed regions represent 29 .5% of the total weights in the decision function. Abbreviations: Inf: Inferior; L: Left; Med: Medial; Mid: Middle; Oper: Opercularis, Orb: Orbital; Post: Posterior; R: Right; Sup: Superior; Supp: Supplementary; % NW(ROI): Percentage of the total normalized weights that each anatomical region explains.

Table D: Measures of agreement between actual and decoded clinical scores for PGBI-10M at screen and closest to scan, based on functional neuroimaging scans (win and loss blocks) using the standard (whole-brain) template from the Pattern Recognition for Neuroimaging Toolbox (PRoNTo, http://www.mlnl.cs.ucl.ac.uk/pronto/).

|  | **Controlled for medication** | | **Not-controlled for medication** | |
| --- | --- | --- | --- | --- |
| **Measures** | **r (*p*-value)** | **MSE (p-value)** | **r (*p*-value)** | **MSE (*p*-value)** |
| **Leave-one-out cross-validation (Win blocks)** | | | | |
| **PGBI-10M at screen** | **0.46 (0.002)** | **31.10 (0.002)** | **0.36 (0.003)** | **34.87 (0.003)** |
| **PGBI-10M closest to scan** | **-0.02 (0.45)** | **33.14 (0.27)** | **0.18 (0.09)** | **28.80 (0.05)** |
| **Four-fold cross-validation (Win blocks)** | | | | |
| **PGBI-10M at screen** | **0.39 (0.004)** | **35.52 (0.003)** | **0.35 (0.009)** | **34.95 (0.002)** |
| **PGBI-10M closest to scan** | **-0.04 (0.54)** | **47.49 (0.91)** | **0.17 (0.11)** | **29.34 (0.07)** |
| **Leave-one-out cross-validation (Loss blocks)** | | | | |
| **PGBI-10M at screen** | **0.39 (0.005)** | **33.40 (0.004)** | **0.31 (0.01)** | **36.34 (0.008)** |
| **PGBI-10M closest to scan** | **-0.11 (0.62)** | **35.19 (0.39)** | **0.10 (0.20)** | **30.90 (0.10)** |
| **Four-fold cross-validation (Loss blocks)** | | | | |
| **PGBI-10M at screen** | **0.34 (0.02)** | **38.44 (0.02)** | **0.25 (0.02)** | **38.61(0.01)** |
| **PGBI-10M closest to scan** | **-0.10 (0.69)** | **55.18 (0.94)** | **0.16 (0.11)** | **29.80 (0.06)** |

Abbreviations: PGBI-10M=Parent General Behavior Inventory 10 Item Mania scale; r=Pearson’s correlation value, MSE=mean squared error.

Table E: Complete list of brain regions ranked according to the normalized weights (NW) to decode PGBI-10M at screen based on pattern of brain activation to win blocks using a leave-one-out cross-validation framework.

| **Rank** | **Brain Regions** | **% NW(ROI)** |
| --- | --- | --- |
| 1 | Frontal Inf Oper L | 1.9 |
| 2 | Cerebelum 4 5 L | 1.7 |
| 3 | Precuneus L | 1.7 |
| 4 | Parietal Inf R | 1.7 |
| 5 | Parietal Sup R | 1.7 |
| 6 | Frontal Inf Orb L | 1.7 |
| 7 | Parietal Inf L | 1.6 |
| 8 | Cerebelum 3 R | 1.6 |
| 9 | Insula L | 1.6 |
| 10 | Supp Motor Area R | 1.5 |
| 11 | Calcarine L | 1.4 |
| 12 | Temporal Pole Sup R | 1.4 |
| 13 | Parietal Sup L | 1.4 |
| 14 | Rectus R | 1.3 |
| 15 | Frontal Mid Orb R | 1.3 |
| 16 | Olfactory L | 1.3 |
| 17 | Fusiform L | 1.2 |
| 18 | Cerebelum Crus1 L | 1.2 |
| 19 | Frontal Mid Orb L | 1.2 |
| 20 | SupraMarginal L | 1.2 |
| 21 | Cerebelum 6 L | 1.2 |
| 22 | Rectus L | 1.2 |
| 23 | Frontal Inf Tri L | 1.2 |
| 24 | Cingulum Ant L | 1.2 |
| 25 | Frontal Mid Orb R | 1.2 |
| 26 | Frontal Sup Medial L | 1.2 |
| 27 | Olfactory R | 1.1 |
| 28 | Cerebelum 4 5 R | 1.1 |
| 29 | Cingulum Post R | 1.1 |
| 30 | Pallidum L | 1.1 |
| 31 | Temporal Pole Mid L | 1.1 |
| 32 | Temporal Pole Sup L | 1.1 |
| 33 | Occipital Mid R | 1.1 |
| 34 | Frontal Inf Tri R | 1.1 |
| 35 | Thalamus L | 1.1 |
| 36 | Occipital Sup L | 1.1 |
| 37 | Angular R | 1.1 |
| 38 | Caudate L | 1.0 |
| 39 | Temporal Sup L | 1.0 |
| 40 | Occipital Mid L | 1.0 |
| 41 | Frontal Inf Orb R | 1.0 |
| 42 | Lingual L | 1.0 |
| 43 | Frontal Mid R | 1.0 |
| 44 | Frontal Mid L | 1.0 |
| 45 | Temporal Sup R | 1.0 |
| 46 | Frontal Sup Medial R | 1.0 |
| 47 | Temporal Inf R | 1.0 |
| 48 | Hippocampus R | 1.0 |
| 49 | Vermis 3 | 1.0 |
| 50 | Frontal Sup L | 1.0 |
| 51 | Angular L | 0.9 |
| 52 | Calcarine R | 0.9 |
| 53 | Cingulum Ant R | 0.9 |
| 54 | Frontal Inf Oper R | 0.9 |
| 55 | Supp Motor Area L | 0.9 |
| 56 | Temporal Mid L | 0.9 |
| 57 | Occipital Sup R | 0.9 |
| 58 | Cingulum Post L | 0.9 |
| 59 | Fusiform R | 0.9 |
| 60 | Cuneus R | 0.9 |
| 61 | Frontal Mid Orb L | 0.9 |
| 62 | Rolandic Oper L | 0.9 |
| 63 | Cingulum Mid L | 0.9 |
| 64 | Vermis 6 | 0.9 |
| 65 | Vermis 7 | 0.8 |
| 66 | Insula R | 0.8 |
| 67 | Cerebelum 6 R | 0.8 |
| 68 | Frontal Sup R | 0.8 |
| 69 | Frontal Sup Orb L | 0.8 |
| 70 | ParaHippocampal R | 0.8 |
| 71 | Occipital Inf L | 0.8 |
| 72 | Precentral R | 0.8 |
| 73 | Heschl R | 0.8 |
| 74 | Precuneus R | 0.8 |
| 75 | Temporal Mid R | 0.8 |
| 76 | Postcentral R | 0.8 |
| 77 | Frontal Sup Orb R | 0.8 |
| 78 | Paracentral Lobule L | 0.7 |
| 79 | Cingulum Mid R | 0.7 |
| 80 | Postcentral L | 0.7 |
| 81 | Rolandic Oper R | 0.7 |
| 82 | SupraMarginal R | 0.7 |
| 83 | Precentral L | 0.7 |
| 84 | Cuneus L | 0.7 |
| 85 | Lingual R | 0.7 |
| 86 | Paracentral Lobule R | 0.7 |
| 87 | ParaHippocampal L | 0.7 |
| 88 | Amygdala L | 0.6 |
| 89 | Vermis 4 5 | 0.6 |
| 90 | Putamen R | 0.6 |
| 91 | Temporal Inf L | 0.6 |
| 92 | Cerebelum 7b L | 0.6 |
| 93 | Amygdala R | 0.6 |
| 94 | Pallidum R | 0.6 |
| 95 | Putamen L | 0.6 |
| 96 | Heschl L | 0.6 |
| 97 | Occipital Inf R | 0.6 |
| 98 | Thalamus R | 0.5 |
| 99 | Caudate R | 0.5 |

Abbreviations: Inf: Inferior; L: Left; Med: Medial; Mid: Middle; Oper: Opercularis, Orb: Orbital; Post: Posterior; R: Right; Sup: Superior; Supp: Supplementary; Tri: Triangularis, Ant: Anterior

Table F: Complete list of brain regions ranked according to the normalized weights (NW) to decode PGBI-10M at screen based on pattern of brain activation to loss blocks using a leave-one-out cross-validation framework.

| **Rank** | **Brain Regions** | **% NW(ROI)** |
| --- | --- | --- |
| 1 | Cerebelum 4 5 L | 2.2 |
| 2 | Frontal Inf Oper L | 2.1 |
| 3 | Parietal Inf L | 2.0 |
| 4 | Cerebelum 3 R | 1.6 |
| 5 | Supp Motor Area R | 1.6 |
| 6 | Parietal Sup R | 1.6 |
| 7 | Frontal Mid Orb R | 1.5 |
| 8 | Frontal Inf Orb L | 1.5 |
| 9 | Cingulum Post R | 1.4 |
| 10 | Parietal Inf R | 1.4 |
| 11 | Temporal Sup L | 1.3 |
| 12 | Frontal Mid Orb R | 1.3 |
| 13 | Cerebelum 6 L | 1.3 |
| 14 | Rectus L | 1.3 |
| 15 | Frontal Inf Tri L | 1.3 |
| 16 | Parietal Sup L | 1.3 |
| 17 | Precuneus L | 1.3 |
| 18 | Cerebelum 4 5 R | 1.3 |
| 19 | Temporal Pole Sup R | 1.2 |
| 20 | Olfactory R | 1.2 |
| 21 | Olfactory L | 1.2 |
| 22 | Frontal Inf Tri R | 1.2 |
| 23 | Occipital Sup L | 1.2 |
| 24 | Occipital Mid L | 1.2 |
| 25 | Calcarine L | 1.2 |
| 26 | Paracentral Lobule R | 1.1 |
| 27 | Thalamus L | 1.1 |
| 28 | Frontal Mid R | 1.1 |
| 29 | Insula L | 1.1 |
| 30 | Frontal Mid Orb L | 1.1 |
| 31 | Fusiform L | 1.1 |
| 32 | Frontal Sup Medial L | 1.1 |
| 33 | Lingual L | 1.1 |
| 34 | Supp Motor Area L | 1.0 |
| 35 | Temporal Sup R | 1.0 |
| 36 | Pallidum L | 1.0 |
| 37 | Caudate L | 1.0 |
| 38 | Frontal Mid L | 1.0 |
| 39 | SupraMarginal L | 1.0 |
| 40 | Angular L | 1.0 |
| 41 | Temporal Pole Mid L | 1.0 |
| 42 | Cerebelum Crus1 L | 1.0 |
| 43 | Temporal Pole Sup L | 1.0 |
| 44 | Temporal Inf R | 1.0 |
| 45 | Occipital Mid R | 1.0 |
| 46 | Rectus R | 1.0 |
| 47 | Rolandic Oper L | 1.0 |
| 48 | ParaHippocampal R | 1.0 |
| 49 | Hippocampus R | 1.0 |
| 50 | Cingulum Ant R | 1.0 |
| 51 | Fusiform R | 1.0 |
| 52 | Cingulum Ant L | 0.9 |
| 53 | Frontal Sup L | 0.9 |
| 54 | Angular R | 0.9 |
| 55 | Frontal Inf Orb R | 0.9 |
| 56 | Frontal Mid Orb L | 0.9 |
| 57 | Cuneus L | 0.9 |
| 58 | Cuneus R | 0.9 |
| 59 | Frontal Inf Oper R | 0.9 |
| 60 | Cingulum Post L | 0.9 |
| 61 | Frontal Sup Orb L | 0.9 |
| 62 | Precentral R | 0.8 |
| 63 | Vermis 4 5 | 0.8 |
| 64 | Cingulum Mid L | 0.8 |
| 65 | Temporal Mid L | 0.8 |
| 66 | Frontal Sup R | 0.8 |
| 67 | Frontal Sup Medial R | 0.8 |
| 68 | Occipital Sup R | 0.8 |
| 69 | Frontal Sup Orb R | 0.8 |
| 70 | ParaHippocampal L | 0.8 |
| 71 | Paracentral Lobule L | 0.8 |
| 72 | Calcarine R | 0.8 |
| 73 | Insula R | 0.8 |
| 74 | Putamen L | 0.8 |
| 75 | Lingual R | 0.8 |
| 76 | Precuneus R | 0.8 |
| 77 | Postcentral L | 0.8 |
| 78 | Precentral L | 0.8 |
| 79 | Vermis 6 | 0.8 |
| 80 | Putamen R | 0.8 |
| 81 | Heschl R | 0.7 |
| 82 | Postcentral R | 0.7 |
| 83 | SupraMarginal R | 0.7 |
| 84 | Pallidum R | 0.7 |
| 85 | Rolandic Oper R | 0.7 |
| 86 | Cingulum Mid R | 0.7 |
| 87 | Occipital Inf L | 0.7 |
| 88 | Amygdala L | 0.7 |
| 89 | Temporal Mid R | 0.7 |
| 90 | Occipital Inf R | 0.7 |
| 91 | Amygdala R | 0.7 |
| 92 | Cerebelum 7b L | 0.7 |
| 93 | Cerebelum 6 R | 0.6 |
| 94 | Vermis 3 | 0.6 |
| 95 | Temporal Inf L | 0.6 |
| 96 | Thalamus R | 0.6 |
| 97 | Caudate R | 0.6 |
| 98 | Vermis 7 | 0.6 |
| 99 | Heschl L | 0.6 |

Abbreviations: Inf: Inferior; L: Left; Med: Medial; Mid: Middle; Oper: Opercularis, Orb: Orbital; Post: Posterior; R: Right; Sup: Superior; Supp: Supplementary; Tri: Triangularis, Ant: Anterior


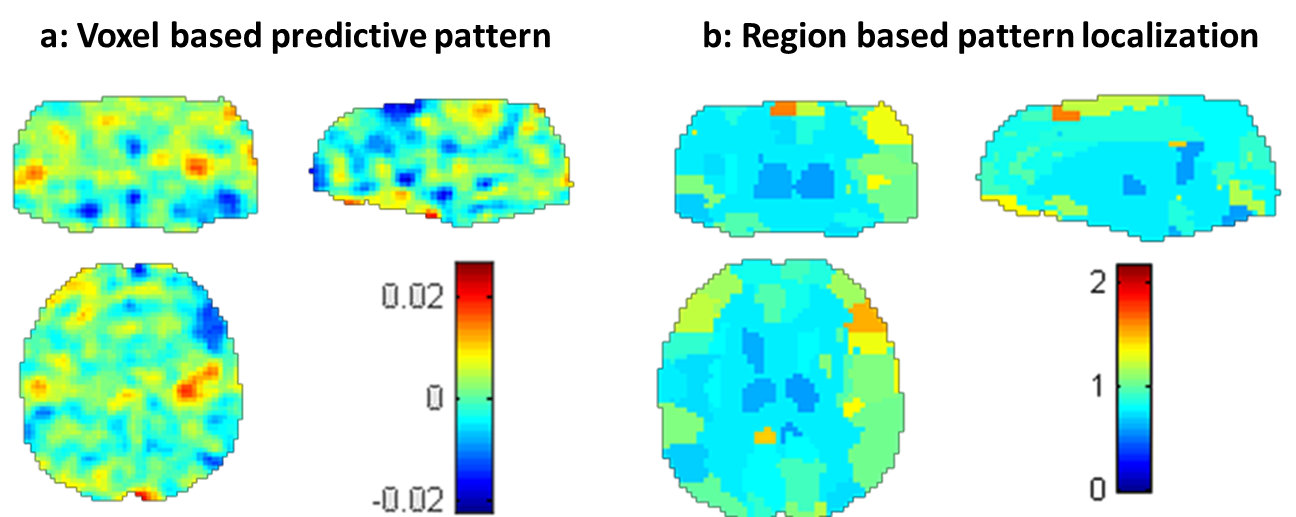


FigA: Maps for decoding PGBI-10M at screen based on patterns of activation to loss blocks using a leave-one-out cross-validation framework. Aa: Voxel-based predictive pattern. The colour bar indicates the weight of the voxels for decoding the clinical score. Ab: Region-based pattern localization map computed from the voxel based predictive pattern displayed in Figure Aa. The colour bar indicates the percentage of the total normalized weights that each anatomically labelled region explains.

**S1 Supporting Information References**

1. Findling RL, Youngstrom EA, Fristad MA, Birmaher B, Kowatch RA, Arnold LE et al (2010) Characteristics of children with elevated symptoms of mania: the Longitudinal Assessment of Manic Symptoms (LAMS) study. J Clin Psychiatry 71: 1664–1672. doi:10.4088/JCP.09m05859yel.Characteristics.

2. Horwitz SMC, Demeter C, Pagano ME, Youngstrom EA, Fristad MA, Arnold LE et al. (2010) Longitudinal Assessment of Manic Symptoms (LAMS) Study: background, design and initial screening results. J Clin Psychiatry 71: 1511–1517. doi:10.4088/JCP.09m05835yel.Longitudinal.

3. Youngstrom E, Meyers O, Demeter C, Youngstrom J, Morello L, Piiparinen R et al. (2005) Comparing diagnostic checklists for pediatric bipolar disorder in academic and community mental health settings. Bipolar Disord 7: 507–517.

4. Youngstrom EA, Frazier TW, Demeter C, Calabrese JR, Findling RL (2008) Developing a Ten Item Mania Scale from the Parent General Behavior Inventory for Children and Adolescents. J Clin Psychiatry 69: 831–839.

5. Bebko G, Bertocci MA, Fournier JC, Hinze AK, Bonar L, et al. (2014) Parsing Dimensional vs Diagnostic Category-Related Patterns of Reward Circuitry Function in Behaviorally and Emotionally Dysregulated Youth in the Longitudinal Assessment of Manic Symptoms Study. JAMA psychiatry 71: 71–80. Available: http://www.ncbi.nlm.nih.gov/pubmed/24285346. Accessed 18 February 2014.
